# Recycling and Reshaping—E3 Ligases and DUBs in the Initiation of T Cell Receptor-Mediated Signaling and Response

**DOI:** 10.3390/ijms23073424

**Published:** 2022-03-22

**Authors:** Clemens Cammann, Nicole Israel, Hortense Slevogt, Ulrike Seifert

**Affiliations:** 1 Friedrich Loeffler-Institute of Medical Microbiology-Virology, University Medicine Greifswald, 17475 Greifswald, Germany; nicole.israel5@googlemail.com; 2 Host Septomics Group, Centre for Innovation Competence (ZIK) Septomics, University Hospital Jena, 07745 Jena, Germany; hortense.slevogt@med.uni-jena.de; 3 Department of Pulmonary Medicine, Hannover Medical School, 30625 Hannover, Germany

**Keywords:** T cell function, T cell receptor signaling, ubiquitination, E3 ligases, deubiquitination, DUBs, SUMO

## Abstract

T cell activation plays a central role in supporting and shaping the immune response. The induction of a functional adaptive immune response requires the control of signaling processes downstream of the T cell receptor (TCR). In this regard, protein phosphorylation and dephosphorylation have been extensively studied. In the past decades, further checkpoints of activation have been identified. These are E3 ligases catalyzing the transfer of ubiquitin or ubiquitin-like proteins to protein substrates, as well as specific peptidases to counteract this reaction, such as deubiquitinating enzymes (DUBs). These posttranslational modifications can critically influence protein interactions by targeting proteins for degradation by proteasomes or mediating the complex formation required for active TCR signaling. Thus, the basic aspects of T cell development and differentiation are controlled by defining, e.g., the threshold of activation in positive and negative selection in the thymus. Furthermore, an emerging role of ubiquitination in peripheral T cell tolerance has been described. Changes in the function and abundance of certain E3 ligases or DUBs involved in T cell homeostasis are associated with the development of autoimmune diseases. This review summarizes the current knowledge of E3 enzymes and their target proteins regulating T cell signaling processes and discusses new approaches for therapeutic intervention.

## 1. Ubiquitination and Deubiquitination—A Basic Introduction

**Figure 1:**
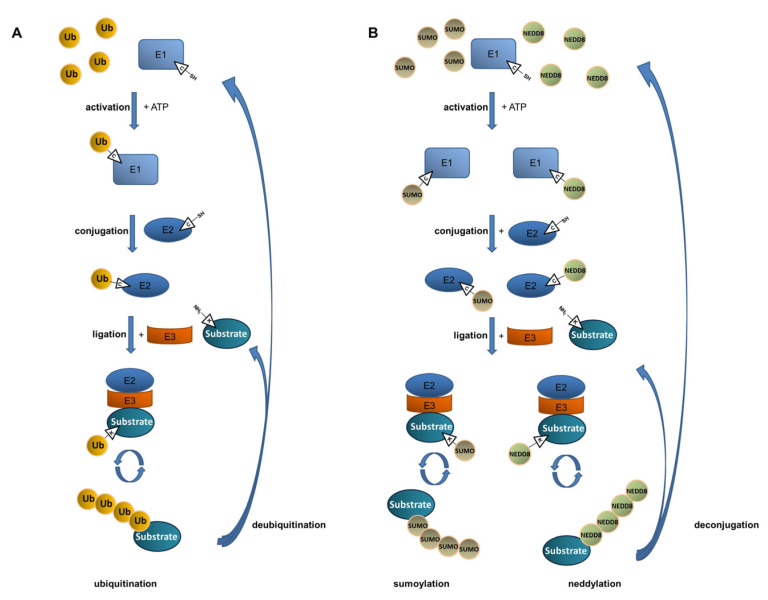
The small modifiers ubiquitin, SUMO, and NEDD8 share the same conjugation mechanism. In the first step, a thioester intermediate is formed between an E1-activating enzyme and ubiquitin (**A**), SUMO, or NEDD8 (**B**) under the consumption of ATP. Subsequently, ubiquitin and ubiquitin-like modifiers are transferred to the E2-conjugating enzyme, forming another thioester linkage. In the final step, the small modifiers are bound to a lysine (K) residue of the substrate by an E3 ligase forming an isopeptide bond. Multiple enzymatic cascades result in elongation of the chains of ubiquitin, SUMO, or NEDD8. Deconjugating enzymes such as DUBs, SUMO proteases, and NEDD8 isopeptidases are able to cleave off the modifications again.

**Figure 2:**
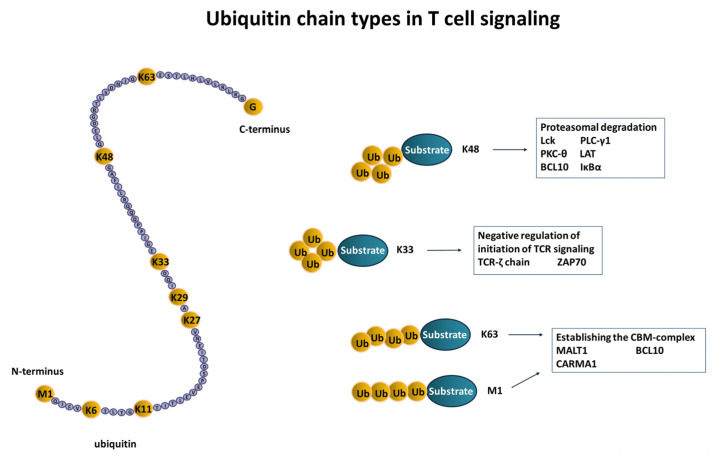
Polyubiquitin chain types in T cell signaling. When protein substrates are polyubiquitinated, isopeptide linkages are formed between a C-terminal glycine (G) of the incoming ubiquitin and the ε-amino group of a lysine (K) residue within the ubiquitin that is attached to the substrate. Therefore, specific polyubiquitin chain linkages can be formed with all seven lysines and the N-terminal methionine of ubiquitin, leading to distinct functions. Depicted here are proteins of the TCR-signaling cascade where the type of ubiquitin chain linkage is known.

Ubiquitination is a posttranslational modification that is involved in almost every cellular process. The most prominent function is the targeting of protein substrates for their degradation by the proteasome to maintain cellular protein homeostasis [1,2,3]. Moreover, ubiquitination can serve many nonproteolytic functions like the regulation of protein kinase signaling, DNA damage response, intracellular trafficking, and transcription and translation [4]. Ubiquitination is mediated by the transfer of the highly conserved 76 amino -acid protein ubiquitin to a target protein. This process results in either monoubiquitinated proteins when a single ubiquitin moiety is transferred or multi-monoubiquitination when monoubiquitination occurs at multiple sites within the protein substrate. Monoubiquitination is the most abundant modification that plays a crucial role in chromatin regulation, protein sorting, and trafficking [5]. Moreover, several defined mono-ubiquitinated proteins are targeted for proteasomal degradation [6]. The repeated addition of ubiquitin moieties to the target protein results in ubiquitin chains of different lengths, namely polyubiquitination. Ubiquitin chains are assembled in a three-step enzymatic process (Figure 1A). Initially, ubiquitin is activated by E1 ubiquitin-activating enzymes through adenylation of the C-terminus under consumption of ATP. The activated ubiquitin is subsequently transferred to a cysteine residue of an E2 ubiquitin-conjugating enzyme with the formation of a thioester bond. By forming a complex with a specific E3 ubiquitin ligase and its substrate, the ubiquitin moiety is covalently bound to a specific lysine residue in the target protein. However, in rare cases, the amino acids threonine, serine, or cysteine were recently identified as targets for ubiquitination as well [7,8,9]. Up to now, three types of E3 ligases could be structurally distinguished, resulting in different enzymatic reactions. E3 ligases containing a really interesting new gene (RING) domain mediate the transfer of ubiquitin by the simultaneous binding of the E2 enzyme and the substrate [10]. Another structural prerequisite is homologous with the E6-associated protein C-terminus (HECT) domain in HECT domain ligases, which contain a catalytically active cysteine residue binding the ubiquitin by forming an intermediary thioester bond and mediating a subsequent transfer of the ubiquitin onto the substrate [11]. The RING-Between-RING (RBR)-type E3 ligases utilize both mechanisms by binding the E2 enzyme to a RING domain, which transfers the ubiquitin to a RING-like domain, forming a thioester intermediate. This entire approach ends in a polyubiquitin chain that contains ubiquitin moieties linked through one of the seven internal ubiquitin lysine (K) residues (which are K6, K11, K27, K29, K33, K48, and K63) or through the ubiquitin amino terminal Met1 residue (Figure 2). The most prominent chain types observed in eukaryotic cells are the K48- and K63-linked chains; however, all possible linkage types have been detected so far [12]. The formation of K48 ubiquitin chains is crucial to target proteins for degradation by 26S proteasomes [4]. K63, K33, and M1 linear ubiquitin chain formation can be detected in modulating protein interactions and protein complex formation to maintain signal transduction processes, e.g., in the NF-κB pathway [13,14]. 

In contrast to the formation of homotypic ubiquitin chains containing only one linkage type, ubiquitin moieties in a chain can be ubiquitinated at multiple K-residues, forming so-called branched structures. Although recent studies revealed that a variety of K11/K48 or K48/K63-branched ubiquitin chains exists, many questions regarding the assembly, recognition, and the potential role of these chains remain [15]. The formation of ubiquitin chains can be reversed by specific deubiquitinating enzymes (DUBs), which hydrolyze the isopeptide bonds between ubiquitin moieties from the distal end of a chain or by cleaving entire chains by breaking the bond between the proximal ubiquitin and the substrate [16,17]. Similar to ubiquitin E3 ligases, most of the investigated DUBs have been shown to be highly linkage-specific enzymes [18]. Next to their role to generate and recycle free ubiquitin monomers, DUBs antagonize the ubiquitination of proteins to reverse possible ubiquitin signals, e.g., rescue proteins targeted for degradation. About 100 different DUBs are known to be expressed in mammalian cells. For most of them, a specific target or function is currently unknown.

## 2. Beyond Ubiquitination—Ubiquitin-like Proteins

In addition to ubiquitin, several ubiquitin-like proteins, such as the small ubiquitin-like modifier (SUMO) and the neuronal precursor cell-expressed developmentally downregulated protein 8 (NEDD8), are involved in various cellular processes. To fulfill their function, ubiquitin-related modifiers are transferred to target proteins (sumoylation or neddylation) and impact their stability, activity, and interactions in cellular signaling pathways. Sumoylation events have been originally detected in the nucleus, playing an important role in major nuclear processes. Especially signal transduction, stress responses, protein stability, and the cell cycle are regulated by sumoylation [19,20], whereas cell cycle progression, metabolism, immunity, and tumorigenesis are affected by neddylation [21].

SUMOs are a family of small proteins that are expressed as the three major isoforms SUMO-1, SUMO-2, and SUMO-3 in mammals. Although the enzymatic machinery responsible for the covalent coupling of SUMO to lysine residues by E1, E2, and E3 enzymes is comparable to ubiquitination, these enzymes are unique and differ from the ones involved in ubiquitination (Figure 1B) [22,23,24]. In contrast to ubiquitination, there is only a single heterodimeric E1 SUMO-activating enzyme, a single E2 SUMO-conjugating enzyme, and a small number of SUMO-E3 ligases. All three SUMO isoforms can form polymeric chains, resulting in poly-sumoylation [25]. 

Another ubiquitin-like post-translational modification is the binding of the small protein NEDD8 to the lysine residue of substrate proteins. This process is characterized by the activity of a NEDD8-activating enzyme E1, a NEDD8-conjugating enzyme E2, and substrate-specific NEDD8-E3 ligases (Figure 1B) [26,27,28]. The transfer of NEDD8 to target proteins also impacts the protein stability, conformation, and function. Cullin-RING ligases (CRLs), the largest family of multiunit E3 ubiquitin ligases targeting many proteins for proteasomal degradation, are regulated by neddylation [29]. Among the ubiquitin-like proteins, NEDD8 shares the highest structural similarity with ubiquitin. Based on this observation, it can be explained that all currently known E3 ligases capable of transferring NEDD8 to protein targets also function as ubiquitin E3 ligases [30,31]. 

Besides ubiquitination, E3 ligases responsible for the transfer of SUMO or NEDD8 play a role in TCR signaling by modulating key proteins during engagement of the TCR and subsequent intracellular downstream signaling.

## 3. Ubiquitin and Ubiquitin-like Proteins in T-Lymphocytes

Since T cells play a central role in the immune system and are indispensable for maintaining the adaptive cell mediated immunity, T cell signaling, and activation have to be tightly controlled. Like phosphorylation, ubiquitination is a reversible and highly dynamic process and critical for normal T cell function. In this regard, modification of T cell signaling proteins by ubiquitin or ubiquitin-related proteins is responsible not only for the initiation of T cell signaling but also for the termination of T cell activity.

The T cell pool is divided into two populations: T-helper cells (Th) expressing the coreceptor CD4 and cytotoxic T cells (CTLs) expressing CD8. These populations can be further characterized as effector, memory, or regulatory T cells, depending on the expression profile of additional surface markers. The T cell receptor (TCR) is assembled during thymic development, where positive and negative selection processes ensure that only receptors with a weak affinity for self-antigenic peptides bound to the major histocompatibility complex (MHC) exit the thymus to enter the periphery. Afterwards, T cells are constantly recirculating between peripheral lymphoid organs and the bloodstream until they encounter their specific antigen. These antigens of foreign, pathogen, or self-origin are expressed on antigen-presenting cells (APCs) like B cells, dendritic cells, and macrophages as peptide–MHC class I or II complexes. Upon antigen recognition by the appropriate TCR, together with a costimulatory signal by CD28, the adaptive T cell-mediated immune response is initiated [32,33]. Up to now, several posttranslational modifications such as ubiquitination have been shown to contribute to the modulation of the T cell response. In this regard, various ubiquitin chain types that elicit different mechanistic outcomes during these regulation processes are involved at different levels of T cell activation (Figure 2). Moreover, few signaling proteins are modified by SUMO or NEDD8, providing further checkpoints in regulating T cell activity. In contrast, other ubiquitin-like proteins such as interferon-stimulated gene 15 (ISG15), which is important in regulating the innate immune responses [34], ubiquitin-fold modifier (Ufm1), which has a key role regulating ER-stress [35], or human leukocyte antigen-F adjacent transcript 10 (FAT10) play minor roles in T cell activation, according to the current literature.

### 3.1. Initiation of T Cell Signaling

**Figure 3:**
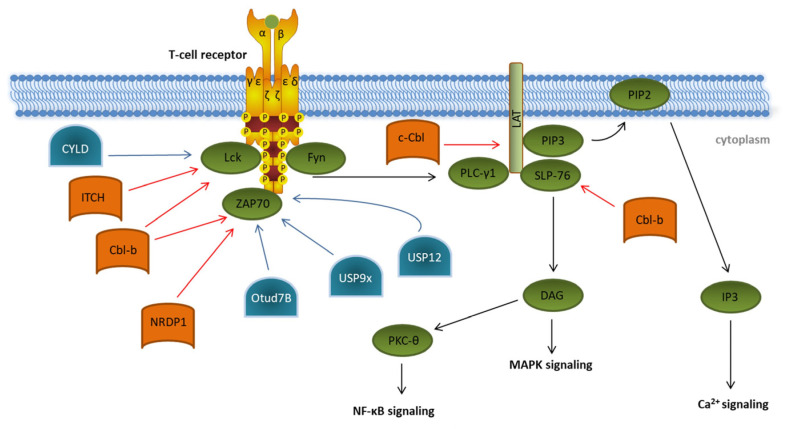
Ubiquitination and deubiquitination in proximal T cell signaling. Overview of known ubiquitin E3 ligases (orange) and deubiquitinating enzymes (blue) involved in regulating the initiation of T cell receptor signaling (green). Details are described in the main text of the manuscript. The T cell signaling pathway is indicated by black arrows. Modifications that dampen T cell receptor signaling are indicated by red arrows. Ubiquitin modifications enhancing T cell signaling are indicated by blue arrows.

The initial step in T cell activation is the conversion of an extracellular signal by binding of an antigen to its specific TCR, which induces an intracellular signaling cascade (Figure 3). This is required to switch the T cell from a resting state to proliferation and differentiation and is characterized by intracellular structural changes such as actin reorganization and metabolic reprogramming [36,37,38]. Structurally, TCR signals are transmitted via the noncovalently associated CD3ζ subunits. These subunits possess intracellular tails containing specific signaling motifs, so-called immunoreceptor tyrosine-based activation motifs (ITAMs). TCR activation results in the phosphorylation of these ITAMs by the two members of the Src-family of protein tyrosine kinases: lymphocyte-specific protein tyrosine kinase Lck and proto-oncogene tyrosine-protein kinase Fyn. Phosphorylated ITAMs provide docking sites for the tandem SH2 domains of the Syk family kinase ζ-chain-associated protein kinase 70 (ZAP70), which is thereby recruited to the plasma membrane and is activated itself via phosphorylation by Lck [39]. Phosphorylated ZAP70 is the key component for further downstream signaling, which leads to phosphorylation of the linker of activation of T cells (LAT) and Src homoly 2 domain-containing leukocyte protein of 76kDa (SLP-76).

In the last decades, several E3 ubiquitin ligases participating in the initiation of T cell signaling have been identified, e.g., the two E3 ligases of the Casitas B-cell Lymphoma (Cbl) protein family, c-Cbl and Cbl-b, and the HECT-type E3 ubiquitin ligase Itchy (ITCH) [40,41,42]. Cbl-b interacts, via its multiple protein-interacting domains, with key TCR signalosome proteins such as Lck, SLP-76, and ZAP70 and targets them for proteasomal degradation (Figure 3) [43,44]. In a cooperative manner with the E3 ligase ITCH, Cbl-b can mediate the K33-linked polyubiquitination of the TCR-ζ chain, which results in reduced ZAP-70 interaction and subsequently decreases downstream phosphorylation [45]. ZAP70 is also targeted by the neuregulin receptor degradation protein-1 (NRDP1)/RNF41 E3 ligase attaching K33-linked ubiquitin chains to ZAP70 (Figure 3) [46]. Both Cbl-b- and NRDP1-dependent ubiquitination of ZAP70 lead to the recruitment of suppressors of T cell signaling 1 and 2 (STS1/2), which dephosphorylate ZAP70, ultimately resulting in an abrogation of downstream signaling. The role of the E3 ligases Cbl-b, ITCH, and NRDP1 in the regulation of T cell activation is further emphasized by data obtained from experiments in KO mice, which showed the hyperproliferation of T cells, reflecting a strong autoimmune phenotype [45,46,47]. Finally, c-Cbl targets the ZAP70 substrate LAT for ubiquitin-mediated degradation, preventing the formation of the LAT complex and therefore abrogating further phosphorylation events downstream of LAT [48].

There are a few deubiquitinating enzymes (DUBs) antagonizing these processes. CYLD, a tumor suppressor in familial cylindromatosis, an autosomal-dominant genetic predisposition to multiple tumors of the skin appendages, interacts with active Lck and promotes the recruitment of active Lck to its substrate Zap70 [49]. Other DUB candidates like the ubiquitin-specific peptidases Usp12 and Usp9x are capable of stabilizing the T cell receptor complex at the plasma membrane, leading to prolonged TCR signaling [50,51]. This was supported by experiments in Usp9x knockout mice pointing to reduced TCR signaling during thymic development, which results in decreased negative selection, ultimately leading to increased numbers of autoreactive T cells [50].

Furthermore, OTUD7B, which is a member of the ovarian tumor (OTU) family of DUBs, deubiquitinates ZAP70, preventing its association with the suppressors of T cell signaling STS1 and STS2, therefore enabling enhanced T cell activation (Figure 3) [52]. This is underlined by the fact that OTUD7B knockout mice are refractory to T cell-mediated autoimmune or inflammatory responses [52]. 

Recently, some signaling proteins that are sumoylated during T cell activation have been identified. The signaling protein phospholipase C-γ1 (PLC-γ1), which is phosphorylated by ZAP70 upon T cell receptor activation, is sumoylated by the Sumo-E3 ligase PIASxβ, which appears to be required for its membrane localization to the LAT complex [53]. In contrast, the desumoylation of PLC-γ1 prevents complex formation and, therefore, blocks further downstream signaling [53]. Moreover, SLP-76, which is also part of the LAT complex, becomes sumoylated rapidly upon T cell stimulation, and this is crucial for activation of the transcription factor nuclear factor of activated T cells (NFAT) [54]. 

Engagement of the TCR and subsequent downstream signaling is accompanied by forming of an immunological synapse at the APC-TCR contact site, resulting in structural and morphological changes bringing surface molecules and intracellular signaling proteins into close proximity. This is important for the successful activation of the T cell signaling pathway [55]. In this regard, TCR-induced sumoylation of the protein kinase C theta (PKC-θ) is essential for the formation of mature immunological synapses and for T cell activation. The desumoylation of PKC-θ blocks its localization to the immune synapse, resulting in dysregulated activation and the proliferation of T cells [56].

Although Cbl-b has been shown to be able to neddylate proteins associated with transforming growth factor-β (TGF-β) signaling, no neddylation has yet been identified in proximal T cell signaling. This is underlined by data showing that application of the neddylation inhibitor MLN4924/Pevonedistad has no effect on the phosphorylation of signaling proteins upon T cell activation [57].

In summary, the initiation of TCR signaling is highly regulated by ubiquitination targeting the TCR-ZAP70 interaction as the main checkpoint. In addition, only a few signaling proteins have been shown to be sumoylated. With respect to the current literature, ubiquitin-like modifiers such as NEDD8, ISG15, or FAT10 are not involved in the proximal T cell signaling cascade.

### 3.2. Transcription Factor Activation in T Cells

The induction of T cell-specific cytokine production, cell proliferation, and differentiation requires the translocation of specific transcription factors into the nucleus.

**Figure 4:**
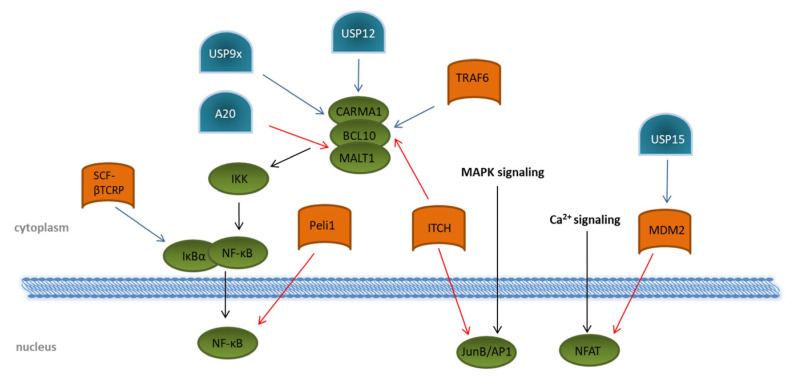
Regulation of transcription-factor activation through ubiquitination and deubiquitination upon T cell stimulation. Overview of known ubiquitin E3 ligases (orange) and deubiquitinating enzymes (blue) involved in the regulation of the transcription factors NF-κB, NFAT, and JunB/AP1 (green). Details are described in the main text of the manuscript. The T cell signaling pathway is indicated by black arrows. Modifications that dampen T cell receptor signaling are indicated by red arrows. Ubiquitin modifications enhancing T cell signaling are indicated by blue arrows.

After triggering the T cell receptor, several signaling cascades downstream of ZAP70 are activated; mainly MAPK pathways via ERK and JNK, modulation of the Ca2+ flux, and activation of PKC-θ lead to the activation of the transcription factors NFAT, the nuclear factor “kappa-light-chain-enhancer” of activated B cells, NF-κB, and JunB/AP-1 (Figure 4). A vast number of transcriptional regulated genes for cytokines, such as IL2, IL6, and IL10; chemokines; and pro-survival proteins, are induced by binding of the NF-κB subunits to the T cell DNA [58,59]. Activation of NF-κB in TCR signaling is strongly dependent on the formation of the CARMA1–BCL10–MALT1 (CBM) complex, consisting of the CARD-containing MAGUK protein 1 (CARMA1), B-cell lymphoma/leukemia 10 (BCL10), and mucosa-associated lymphoid tissue lymphoma translocation protein 1 homolog (MALT1). This, in turn, activates the IKK complex, resulting in proteasomal degradation of the inhibitory protein IκBα induced by the SCF–βTCRP ubiquitin ligase complex (Figure 4) [60,61]. Removal of IκBα allows the active transcription factor NF-κB to translocate into the nucleus [62,63]. Several E3 ligases are involved in regulating the formation of the CBM complex. ITCH attaches K48 ubiquitin chains to BCL10 [64] for its degradation by proteasomes. The DUBs USP9x, as well as USP12, antagonizes this reaction by specifically hydrolyzing K48-linked ubiquitin chains, thereby supporting the CBM complex association and prolonged the activation of NF-κB [65,66]. Augmented activation of downstream kinases, such as the inhibitor of nuclear factor-κB (NF-κB) kinases (IKK) α and β, is mediated by the association of the K63 specific E2 enzyme dimer UBC13/UEV1A with the CBM complex. Together with an E3 ligase, K63 ubiquitin chains are then transferred onto BCL10 and MALT1 [67,68]. Although the involved E3 ligase was identified as tumor necrosis factor (TNF) receptor-associated factor 6 (TRAF6), a specific TRAF6 knockout revealed no impact on NF-κB activation following TCR triggering, suggesting the contribution of other E3 ligases [69]. As a counter-regulation, the DUB A20 is able to remove K63-polyubiquitin chains from the CBM complex protein MALT1, thereby downmodulating TCR-induced NF-κB activity [70]. Interestingly, MALT1 can cleave A20 in a TCR-dependent manner, and this proteolytic cleavage has been suggested to disrupt the ability of A20 to limit TCR downstream signaling via NF-κB inhibition [71]. A20-dependent downregulation of NF-κB-mediated inflammation is further underlined by the fact that NF-κB activity is negatively regulated by A20 overexpression in mice. In addition, patients with an active autoimmune phenotype due to Behcet’s disease reveal a decreased expression of A20 in CD4+ T cells [72].

Direct interaction with the NF-κB subunit c-Rel is mediated by the RING-type E3 ubiquitin protein ligase pellino homolog 1 (PELI1), which attaches K48-linked ubiquitin chains to c-Rel and, thus, initiates its degradation by proteasomes [73]. Although PELI1 is already constitutively expressed in T cells at a steady state, T cell activation further increases its expression, which subsequently interrupts NF-κB-induced gene transcription, thereby preventing autoimmunity [73].

Besides NF-κB, members of the NFAT family, including NFATc1, NFATc2, and NFATc4, and the transcription factor JunB/AP1 are activated upon TCR stimulation by increased Ca^2+^ flux. Both are responsible for the induced transcription of several cytokines, such as IL2, IL4, TNFα, and IFN-γ. Interestingly, they can cooperatively bind DNA, thereby enhancing the DNA-binding and transcriptional activity of each other [74,75]. Based on this observation, the question arises whether the regulation of one transcription factor might affect the activity of the other.

With regard to NFAT, the E3 ligase mouse double minute 2 homolog (MDM2) targets the NFATc2 subunit for degradation by ubiquitination [76]. Interestingly, MDM2 was shown to auto-ubiquitinate itself, leading to K48-linked proteasomal degradation upon T cell activation, which, in turn, amplifies the nuclear accumulation of NFATc2. This is crucial for the induction of cytokines, especially of IFN-γ [76]. The DUB USP15 antagonizes MDM2 ubiquitination by deubiquitination through a zinc finger motif-mediated mechanism, thereby negatively regulating transcription activity of NFAT and differentiation of IFN-γ-producing Th1 effector T cells (Figure 4) [76,77].

Translocation of NFAT to the nucleus is directly influenced by the small ubiquitin-like protein SUMO. The highly sumoylated isoform NFATc1 is translocated to promyelocytic leukemia bodies in the nucleus, leading to the deacetylation of histones and suppression of interleukin-2 gene transcription in vitro [78]. Recently, NFATc1 sumoylation was analyzed in a transgenic mouse model in which the SUMO modification of NFATc1 was blocked. Accordingly, these mice had significantly high IL2 production and enhanced proliferation of regulatory T cells, as well as suppressed IL17 and IFN-γ release [79].

The levels of the transcription factor JunB/AP1 depend on ITCH ubiquitination. ITCH-deficient mice had elevated levels of JunB in the nucleus, which led to increased cytokine production and Th2-type inflammation [80]. Moreover, it has been shown that the sumoylation of JunB is required for the translocation and subsequent expression of T cell-associated cytokine genes like IL2, IL4, and IL10 [79].

Although only a few targets have been determined so far, there is evidence for a regulatory role of neddylation in T cell function. It has been demonstrated that the deficiency of the NEDD8-conjugating enzyme UBC12 leads to diminished proliferation, Th1 and Th2 differentiation, and cytokine production in vitro and in vivo by altered MAPK signaling [81]. In a parasite infection model, neddylation was required for CD4+ T cell proliferation and Th1-cell differentiation and survival, as well as IFN-γ and anti-parasite IgG responses [82]. One explanation could be that the neddylation of cullin-ring ligases is responsible for the degradation of IκBα, thereby influencing the NF-κB pathway. The pharmacological blockade of the entire neddylation by MLN4924/Pevonedistad has been proven to cause an accumulation of IκBα and abrogated translocation of NF-κB into the nucleus in T cells from chronic lymphocytic leukemia [57]. Therefore, it is very likely that the observed effects are due to changes in NF-κB translocation. However, the exact targets have not yet been identified.

Thus, T cell-specific transcription factors fulfill multiple redundant functions, with the disruption of one factor affecting the function of the other. Ubiquitination, sumoylation, and, in part, neddylation are critical processes for regulating transcription factor trafficking and effector functions.

### 3.3. T Cell Development

Mature T cells are constantly recirculating between peripheral lymphoid organs and the bloodstream until they encounter their specific antigen. To ensure that T cells respond to foreign antigens from invading pathogens and simultaneously tolerate self-antigens, T cells have to be educated during thymic development. In the thymus, positive and negative selection processes define the assembled T cell repertoire that only T cell receptors with a weak affinity for self-antigenic peptides bound to the major histocompatibility complex (MHC) are selected. The development and selection of T cells is provided by thymic epithelial cells (TECs) divided in cortical TECs responsible for positive selection and medullary TECs (mTECs) crucial for negative selection [83]. Therefore, mTEC maturation is a critical step in thymic selection, which is regulated by activation of the NF-κB signaling pathway via the TNF receptor superfamily members CD40, receptor activator of NF-κB (RANK), and lymphotoxin β receptor (LTβR) [84,85,86]. Studies on TRAF6 knockout mice, an essential mediator in NF-κB activation that displays E3 ligase activity, have been accompanied with the expression of fewer tissue-specific antigens and compromised mTEC development, leading to an autoimmune phenotype [87]. Moreover, TRAF6 deficiency results in a reduced expression of the autoimmune regulator (AIRE), which is pivotal for negative selection mediating the expression of many tissue-restricted self-antigens. AIRE consists of two plant homeodomains (PHDs), in which PHD1 is speculated to have E3 ligase activity [88]. In the last twenty years, numerous functions of AIRE have been described. Due to its nuclear localization, it binds chromatin and histones and is involved in gene transcription. Furthermore, it is able to enhance alternative mRNA splicing. Altogether, this enables AIRE to regulate the expression of tissue-specific antigens in the mTECs mandatory for negative selection [89].

In addition, the ubiquitin-like protein FAT10 contributes to negative selection. FAT 10 has been shown to be expressed in mTECs [90]. The knockout of FAT10 in mice revealed changes in the T cell repertoire of CD4 and CD8 single positive cells during thymic development, probably due to an altered peptide presentation that influences negative selection [91].

In summary, the regulation of T cell selection processes is crucial for maintaining a functional adaptive immune response. Mutations in specific E3 ligases or DUBs critically affect T cell homeostasis and often lead to alterations in immune responses that ultimately can result in autoimmune diseases and cancer, as well as complications in fighting infectious diseases.

## 4. Role of Ubiquitination in Anergy and Autoimmune Disease

In the last decade, the finding of E3 ligase mutations has been connected to the development of severe autoimmune diseases. For example, mutations in the gene encoding for Cbl-b resulting in reduced Cbl-b function have been associated with type 1 diabetes and multiple sclerosis (MS) [92,93], whereas the aberrant expression of ITCH causes a syndromic multisystem autoimmune disease with acute liver failure [94]. One reason for this observation could be the role of these E3 ligases in inducing T cell anergy. T cell anergy is a crucial peripheral tolerance mechanism preventing the recognition of self-antigens, which can result in autoimmune diseases. Upon the encounter of a self-antigen without a sufficient costimulatory signal, e.g., CD28, or present inhibitory signals, e.g., CTLA4, T cells shut down proliferation and differentiation and remain unresponsive. It was shown that T cells from ITCH- and Cbl-b-deficient mice were hyperproliferative and resistant to anergy induction [42]. Although Cbl-b is required to prevent excessive TCR activation, a recent study revealed that Cbl-b deficiency did not prevent the expression of phenotypic markers of anergy; however, Cbl-b deletion did restore the functional responses to TCR stimulation [95]. Moreover Cbl-b-deficient mice showed similar abnormalities in T cell function as described in patients with MS [96] or patients with systemic lupus erythematosus (SLE) [97]. Although there are currently no pharmacological strategies to enhance the activity of Cbl-b, the development of inhibitors blocking intramolecular inhibitory regions such as the non-phosphorylated N-terminal region can be used to enhance the activity of Cbl-b [98]. Alternatively, the inhibition of Src homology region 2 domain-containing phosphatase-1 (SHP-1), which binds to Cbl-b upon T cell stimulation and abolishes its ubiquitin ligase activity, may enhance Cbl-b activity [99].

On the other hand, the resistance of Cbl-b- or ITCH-deficient T cells to negative signals such as tumor-associated immunosuppressive factors makes them a promising target in cancer therapy. This is underlined by the fact that Cbl-b-deficient mice develop fewer ultraviolet B (UVB)-induced skin malignancies and reject UVB-induced skin tumors [100]. Furthermore, these mice reject transferred TC-1 tumor cells due to the massive infiltration of CD8+ T cells [101]. This makes these E3 ligases valuable targets in treating allograft rejection or to induce immune responses against tumors.

Besides new inhibitors, the modulation of ubiquitination processes by proteolysis targeting chimeras (PROTACS) has been evaluated in recent studies. PROTACS are small linkers that force E3 ligases to ubiquitinate specific substrates and target them to proteasomal degradation. The use of PROTACS introduces a possibility for modulating T cell activity as a new therapeutic approach [102,103]. However, to implement this as a clinical approach, knowledge about the exact interactions between the E3 ligases and their respective substrates is critical.

Additionally, targeting DUBs can also influence T cell responses. For example, ubiquitin-specific protease 4 (USP4) is responsible for Th17-cell differentiation by stabilizing transcription factor RORγT [104]. Inhibiting USP4 could be a valuable target in treating Th17-dependent autoimmune diseases like multiple sclerosis or rheumatoid arthritis. With the development of new DUB-inhibitors [105], this class of enzymes comes into the focus of research as a candidate for further clinical studies [106].

In addition to DUB inhibition, the development of new sumoylation and neddylation inhibitors has increased in recent years. To take advantage of the fact that there is only a single E1 and single E2 enzyme, inhibitors have been developed that strongly abrogate all respective-dependent processes [107,108,109,110]. Currently, they are mainly used in tumor therapy, but it is likely that successful inhibitor strategies can be transferred to other disease patterns in the future. An important basis for this is a more detailed investigation of the role of E3 ligases and deconjugating enzymes/DUBs in refining T cell signaling, which will increase the knowledge regarding disease progression and provide new potential targets for therapeutic intervention.

## 5. Conclusions

Ubiquitination plays a fundamental role in regulating signaling networks and cellular activation, which are crucial to maintaining a functional immune system with a tightly controlled T cell activation. In this review, we highlighted the role of ubiquitin and ubiquitin-like proteins in TCR signaling. The regulation of signaling proteins can be thought of as an amplifier, where phosphorylation and dephosphorylation are reflected in the volume, and ubiquitination and deubiquitination are the equalizers that change the different pitches. Moreover, ubiquitin-like proteins add another layer of regulation either by directly affecting the stability and function of signaling proteins or by interfering with ubiquitination processes. Especially regarding T cell activation, the exact understanding of how different posttranslational modifications fine-tune complex signaling processes in T cells will reveal the key roles of several E3 ligases and DUBs. Furthermore, mutations or changes in the expression level of ubiquitinating enzymes can contribute, on the one hand, to the development of autoimmune diseases. On the other hand, the targeted modulation of E3 enzymes or DUBs can help to enhance T cell proliferation, which may be advantageous for T cell-mediated cancer immunotherapy.

The knowledge of the exact relationship between E3 ligases, their substrates, and the corresponding deubiquitinating/deconjugating enzymes will strengthen the understanding of intracellular pathways and the development of diseases. Interfering with these processes either by developing new inhibitors or PROTACS might be a powerful approach to develop novel therapeutical strategies. 

## Abbreviations

TCR: T cell receptor; DUB: deubiquitinating enzyme; HECT-domain: homologous to the E6-AP carboxyl terminus domain; RING domain: really interesting new gene domain; SUMO: small ubiquitin-like modifier; NEDD8: neuronal precursor cell-expressed developmentally down-regulated protein 8; ZAP70: ζ-chain-associated protein kinase 70; Cbl: casitas B-lineage lymphoma; APC: antigen presenting cell; CBM complex: CARMA1-BCL10-MALT1 complex: CARMA1: caspase-recruitment domain (CARD) membrane-associated guanylate kinase (MAGUK) protein 1; BLC10: B cell lymphoma/leukemia 10; MALT1: mucosa-associated lymphoid tissue lymphoma translocation protein 1 homolog; AIRE: autoimmune regulator.

## References

[1] Hershko A., Ciechanover A. (1998). The ubiquitin system. Annu. Rev. Biochem..

[2] Seifert U., Bialy L.P., Ebstein F., Bech-Otschir D., Voigt A., Schröter F., Prozorovski T., Lange N., Steffen J., Rieger M. (2010). Immunoproteasomes Preserve Protein Homeostasis upon Interferon-Induced Oxidative Stress. Cell.

[3] Colberg L., Cammann C., Greinacher A., Seifert U. (2020). Structure and function of the ubiquitin-proteasome system in platelets. J. Thromb. Haemost..

[4] Komander D., Rape M. (2012). The Ubiquitin Code. Annu. Rev. Biochem..

[5] Husnjak K., Dikic I. (2012). Ubiquitin-Binding Proteins: Decoders of Ubiquitin-Mediated Cellular Functions. Annu. Rev. Biochem..

[6] Braten O., Livneh I., Ziv T., Admon A., Kehat I., Caspi L.H., Gonen H., Bercovich B., Godzik A., Jahandideh S. (2016). Numerous proteins with unique characteristics are degraded by the 26S proteasome following monoubiquitination. Proc. Natl. Acad. Sci. USA.

[7] Ishikura S., Weissman A.M., Bonifacino J.S. (2010). Serine Residues in the Cytosolic Tail of the T-cell Antigen Receptor α-Chain Mediate Ubiquitination and Endoplasmic Reticulum-associated Degradation of the Unassembled Protein. J. Biol. Chem..

[8] Anania V.G., Bustos D.J., Lill J.R., Kirkpatrick D., Coscoy L. (2013). A Novel Peptide-Based SILAC Method to Identify the Posttranslational Modifications Provides Evidence for Unconventional Ubiquitination in the ER-Associated Degradation Pathway. Int. J. Proteom..

[9] Tait S.W., de Vries E., Maas C., Keller A.M., D’Santos C.S., Borst J. (2007). Apoptosis induction by Bid requires unconventional ubiquitination and degradation of its N-terminal fragment. J. Cell Biol..

[10] Metzger M.B., Pruneda J.N., Klevit R.E., Weissman A.M. (2014). RING-type E3 ligases: Master manipulators of E2 ubiquitin-conjugating enzymes and ubiquitination. Biochim. Biophys. Acta.

[11] Metzger M.B., Hristova V.A., Weissman A.M. (2012). HECT and RING finger families of E3 ubiquitin ligases at a glance. J. Cell Sci..

[12] Kim W., Bennett E.J., Huttlin E.L., Guo A., Li J., Possemato A., Sowa M.E., Rad R., Rush J., Comb M.J. (2011). Systematic and Quantitative Assessment of the Ubiquitin-Modified Proteome. Mol. Cell.

[13] Laplantine E., Fontan E., Chiaravalli J., Lopez T., Lakisic G., Véron M., Agou F., Israël A. (2009). NEMO specifically recognizes K63-linked poly-ubiquitin chains through a new bipartite ubiquitin-binding domain. EMBO J..

[14] Ordureau A., Smith H., Windheim M., Peggie M., Carrick E., Morrice N., Cohen P. (2008). The IRAK-catalysed activation of the E3 ligase function of Pellino isoforms induces the Lys63-linked polyubiquitination of IRAK1. Biochem. J..

[15] French M.E., Koehler C.F., Hunter T. (2021). Emerging functions of branched ubiquitin chains. Cell Discov..

[16] Reyes-Turcu F.E., Ventii K.H., Wilkinson K.D. (2009). Regulation and Cellular Roles of Ubiquitin-Specific Deubiquitinating Enzymes. Annu. Rev. Biochem..

[17] Komander D. (2010). Mechanism, Specificity and Structure of the Deubiquitinases. Subcell. Biochem..

[18] Mevissen T.E., Komander D. (2017). Mechanisms of Deubiquitinase Specificity and Regulation. Annu. Rev. Biochem..

[19] Eifler K., Cuijpers S.A.G., Willemstein E., Raaijmakers J., El Atmioui D., Ovaa H., Medema R., Vertegaal A.C.O. (2018). SUMO targets the APC/C to regulate transition from metaphase to anaphase. Nat. Commun..

[20] Enserink J.M. (2015). Sumo and the cellular stress response. Cell Div..

[21] Zou T., Zhang J. (2021). Diverse and pivotal roles of neddylation in metabolism and immunity. FEBS J..

[22] Geiss-Friedlander R., Melchior F. (2007). Concepts in sumoylation: A decade on. Nat. Rev. Mol. Cell Biol..

[23] Gareau J.R., Lima C.D. (2010). The SUMO pathway: Emerging mechanisms that shape specificity, conjugation and recognition. Nat. Rev. Mol. Cell Biol..

[24] Pichler A., Fatouros C., Lee H., Eisenhardt N. (2017). SUMO conjugation—A mechanistic view. Biomol. Concepts.

[25] Hendriks I.A., Lyon D., Young C., Jensen L.J., Vertegaal A.C.O., Nielsen M.L. (2017). Site-specific mapping of the human SUMO proteome reveals co-modification with phosphorylation. Nat. Struct. Mol. Biol..

[26] Pan Z.-Q., Kentsis A., Dias D.C., Yamoah K., Wu K. (2004). Nedd8 on cullin: Building an expressway to protein destruction. Oncogene.

[27] Huang D.T., Ayrault O., Hunt H.W., Taherbhoy A.M., Duda D.M., Scott D.C., Borg L.A., Neale G., Murray P.J., Roussel M.F. (2009). E2-RING Expansion of the NEDD8 Cascade Confers Specificity to Cullin Modification. Mol. Cell.

[28] Li J., Zou J., Littlejohn R., Liu J., Su H. (2020). Neddylation, an Emerging Mechanism Regulating Cardiac Development and Function. Front. Physiol..

[29] Duda D.M., Borg L.A., Scott D.C., Hunt H.W., Hammel M., Schulman B.A. (2008). Structural Insights into NEDD8 Activation of Cullin-RING Ligases: Conformational Control of Conjugation. Cell.

[30] Baek K., Scott D.C., Schulman B.A. (2021). NEDD8 and ubiquitin ligation by cullin-RING E3 ligases. Curr. Opin. Struct. Biol..

[31] Santonico E. (2019). New Insights into the Mechanisms Underlying NEDD8 Structural and Functional Specificities. Ubiquitin Proteasome System—Current Insights into Mechanism Cellular Regulation and Disease.

[32] Von Andrian U.H., Mackay C.R. (2000). T-Cell Function and Migration—Two Sides of the Same Coin. N. Engl. J. Med..

[33] Mescher M.F., Curtsinger J.M., Agarwal P., Casey K.A., Gerner M., Hammerbeck C.D., Popescu F., Xiao Z. (2006). Signals required for programming effector and memory development by CD8 + T cells. Immunol. Rev..

[34] Zhang M., Li J., Yan H., Huang J., Wang F., Liu T., Zeng L., Zhou F. (2021). ISGylation in Innate Antiviral Immunity and Pathogen Defense Responses: A Review. Front. Cell Dev. Biol..

[35] Lemaire K., Moura R.F., Granvik M., Igoillo-Esteve M., Hohmeier H.E., Hendrickx N., Newgard C.B., Waelkens E., Cnop M., Schuit F. (2011). Ubiquitin Fold Modifier 1 (UFM1) and Its Target UFBP1 Protect Pancreatic Beta Cells from ER Stress-Induced Apoptosis. PLoS ONE.

[36] Hwang J.-R., Byeon Y., Kim D., Park S.-G. (2020). Recent insights of T cell receptor-mediated signaling pathways for T cell activation and development. Exp. Mol. Med..

[37] Cammann C., Schraven B., Lindquist J.A. (2013). T cell Metabolism—Regulating Energy. J. Clin. Cell. Immunol..

[38] Cammann C., Rath A., Reichl U., Lingel H., Brunner-Weinzierl M., Simeoni L., Schraven B., Lindquist J.A. (2016). Early changes in the metabolic profile of activated CD8(+) T cells. BMC Cell Biol..

[39] Chan A.C., Iwashima M., Turck C.W., Weiss A. (1992). ZAP-70: A 70 kd protein-tyrosine kinase that associates with the TCR zeta chain. Cell.

[40] Bachmaier K., Krawczyk C.M., Kozieradzki I., Kong Y.-Y., Sasaki T., Oliveira-Dos-Santos A., Mariathasan S., Bouchard D., Wakeham A., Itie A. (2000). Negative regulation of lymphocyte activation and autoimmunity by the molecular adaptor Cbl-b. Nature.

[41] Chiang Y.J., Kole H.K., Brown K., Naramura M., Fukuhara S., Hu R.-J., Jang I.K., Gutkind J.S., Shevach E.M., Gu H. (2000). Cbl-b regulates the CD28 dependence of T-cell activation. Nature.

[42] Venuprasad K. (2010). Cbl-b and Itch: Key Regulators of Peripheral T-cell Tolerance. Cancer Res..

[43] Rao N., Miyake S., Reddi A.L., Douillard P., Ghosh A.K., Dodge I.L., Zhou P., Fernandes N.D., Band H. (2002). Negative regulation of Lck by Cbl ubiquitin ligase. Proc. Natl. Acad. Sci. USA.

[44] Zhang Z., Elly C., Qiu L., Altman A., Liu Y.-C. (1999). A direct interaction between the adaptor protein Cbl-b and the kinase Zap-70 induces a positive signal in T cells. Curr. Biol..

[45] Huang H., Jeon M.-S., Liao L., Yang C., Elly C., Yates J.R., Liu Y.-C. (2010). K33-Linked Polyubiquitination of T Cell Receptor-Zeta Regulates Proteolysis-Independent T Cell Signaling. Immunity.

[46] Yang M., Chen T., Li X., Yu Z., Tang S., Wang C., Gu Y., Liu Y., Xu S., Li W. (2015). K33-linked polyubiquitination of Zap70 by Nrdp1 controls CD8+ T cell activation. Nat. Immunol..

[47] Zhang J., Bárdos T., Li D., Gál I., Vermes C., Xu J., Mikecz K., Finnegan A., Lipkowitz S., Glant T.T. (2002). Cutting edge: Regulation of T cell activation threshold by CD28 costimulation through targeting Cbl-b for ubiquitination. J. Immunol..

[48] Balagopalan L., Barr V.A., Sommers C.L., Barda-Saad M., Goyal A., Isakowitz M.S., Samelson L.E. (2007). c-Cbl-Mediated Regulation of LAT-Nucleated Signaling Complexes. Mol. Cell. Biol..

[49] Reiley W.W., Zhang M., Jin W., Losiewicz M., Donohue K.B., Norbury C.C., Sun S.-C. (2006). Regulation of T cell development by the deubiquitinating enzyme CYLD. Nat. Immunol..

[50] Naik E., Webster J.D., Devoss J., Liu J., Suriben R., Dixit V.M. (2014). Regulation of proximal T cell receptor signaling and tolerance induction by deubiquitinase Usp9X. J. Exp. Med..

[51] Jahan A.S., Lestra M., Swee L.K., Fan Y., Lamers M.M., Tafesse F.G., Theile C.S., Spooner E., Bruzzone R., Ploegh H.L. (2016). Usp12 stabilizes the T-cell receptor complex at the cell surface during signaling. Proc. Natl. Acad. Sci. USA.

[52] Hu H., Wang H., Xiao Y., Jin J., Chang J.-H., Zou Q., Xie X., Cheng X., Sun S.-C. (2016). Otud7b facilitates T cell activation and inflammatory responses by regulating Zap70 ubiquitination. J. Exp. Med..

[53] Wang Q.-L., Liang J.-Q., Gong B.-N., Xie J.-J., Yi Y.-T., Lan X., Li Y. (2019). T Cell Receptor (TCR)-Induced PLC-γ1 Sumoylation via PIASxβ and PIAS3 SUMO E3 Ligases Regulates the Microcluster Assembly and Physiological Function of PLC-γ1. Front. Immunol..

[54] Xiong Y., Yi Y., Wang Y., Yang N., Rudd C.E., Liu H. (2019). Ubc9 Interacts with and SUMOylates the TCR Adaptor SLP-76 for NFAT Transcription in T Cells. J. Immunol..

[55] Dustin M.L. (2014). The Immunological Synapse. Cancer Immunol. Res..

[56] Wang X.-D., Gong Y., Chen Z.-L., Gong B.-N., Xie J.-J., Zhong C.-Q., Wang Q.-L., Diao L.-H., Xu A., Han J. (2015). TCR-induced sumoylation of the kinase PKC-θ controls T cell synapse organization and T cell activation. Nat. Immunol..

[57] Best S., Lam V., Liu T., Bruss N., Kittai A., Danilova O.V., Murray S., Berger A., Pennock N.D., Lind E.F. (2021). Immunomodulatory effects of pevonedistat, a NEDD8-activating enzyme inhibitor, in chronic lymphocytic leukemia-derived T cells. Leukemia.

[58] Oeckinghaus A., Ghosh S. (2009). The NF-kappaB Family of Transcription Factors and Its Regulation. Cold Spring Harb. Perspect. Biol..

[59] Gerondakis S., Siebenlist U. (2010). Roles of the NF-kappaB Pathway in Lymphocyte Development and Function. Cold Spring Harb. Perspect. Biol..

[60] Kanarek N., Ben-Neriah Y. (2012). Regulation of NF-κB by ubiquitination and degradation of the IκBs. Immunol. Rev..

[61] Yaron A., Hatzubai A., Davis M., Lavon I., Amit S., Manning A.M., Andersen J.S., Mann M., Mercurio F., Ben-Neriah Y. (1998). Identification of the receptor component of the IκBα-ubiquitin ligase. Nature.

[62] Ruefli-Brasse A.A., French D.M., Dixit V.M. (2003). Regulation of NF-κB-Dependent Lymphocyte Activation and Development by Paracaspase. Science.

[63] Egawa T., Albrecht B., Favier B., Sunshine M.J., Mirchandani K., O’Brien W., Thome M., Littman D.R. (2003). Requirement for CARMA1 in Antigen Receptor-Induced NF-κB Activation and Lymphocyte Proliferation. Curr. Biol..

[64] Scharschmidt E., Wegener E., Heissmeyer V., Rao A., Krappmann D. (2004). Degradation of Bcl10 Induced by T-Cell Activation Negatively Regulates NF-κB Signaling. Mol. Cell. Biol..

[65] Park Y., Jin H.-S., Liu Y.-C. (2013). Regulation of T cell function by the ubiquitin-specific protease USP9X via modulating the Carma1-Bcl10-Malt1 complex. Proc. Natl. Acad. Sci. USA.

[66] Fu Y., Wang P., Zhao J., Tan Y., Sheng J., He S., Du X., Huang Y., Yang Y., Li J. (2021). USP12 promotes CD4(+) T cell responses through deubiquitinating and stabilizing BCL10. Cell Death Differ..

[67] Sun L., Deng L., Ea C.-K., Xia Z.-P., Chen Z.J. (2004). The TRAF6 Ubiquitin Ligase and TAK1 Kinase Mediate IKK Activation by BCL10 and MALT1 in T Lymphocytes. Mol. Cell.

[68] Zhou H., Wertz I., O’Rourke K., Ultsch M., Seshagiri S., Eby M., Xiao W., Dixit V.M. (2003). Bcl10 activates the NF-κB pathway through ubiquitination of NEMO. Nature.

[69] King C.G., Kobayashi T., Cejas P.J., Kim T., Yoon K., Kim G.K., Chiffoleau E., Hickman S.P., Walsh P.T., Turka L.A. (2006). TRAF6 is a T cell-intrinsic negative regulator required for the maintenance of immune homeostasis. Nat. Med..

[70] Düwel M., Welteke V., Oeckinghaus A., Baens M., Kloo B., Ferch U., Darnay B.G., Ruland J., Marynen P., Krappmann D. (2009). A20 Negatively Regulates T Cell Receptor Signaling to NF-κB by Cleaving Malt1 Ubiquitin Chains. J. Immunol..

[71] Coornaert B., Baens M., Heyninck K., Bekaert T., Haegman M., Staal J., Sun L., Chen Z.J., Marynen P., Beyaert R. (2008). T cell antigen receptor stimulation induces MALT1 paracaspase–mediated cleavage of the NF-κB inhibitor A20. Nat. Immunol..

[72] Hu J., Yi S., Wang C., Zhang Y., Tang J., Huang X., Yang L., Yang J., Li H. (2021). A20 Inhibits Intraocular Inflammation in Mice by Regulating the Function of CD4+T Cells and RPE Cells. Front. Immunol..

[73] Chang M., Jin W., Chang J.-H., Xiao Y., Brittain G.C., Yu J., Zhou X., Wang Y.-H., Cheng X., Li P. (2011). The ubiquitin ligase Peli1 negatively regulates T cell activation and prevents autoimmunity. Nat. Immunol..

[74] Chinenov Y., Kerppola T.K. (2001). Close encounters of many kinds: Fos-Jun interactions that mediate transcription regulatory specificity. Oncogene.

[75] Li B., Tournier C., Davis R.J., Flavell R.A. (1999). Regulation of IL-4 expression by the transcription factor JunB during T helper cell differentiation. EMBO J..

[76] Zou Q., Jin J., Hu H., Li H.S., Romano S., Xiao Y., Nakaya M., Zhou X., Cheng X., Yang P. (2014). USP15 stabilizes MDM2 to mediate cancer-cell survival and inhibit antitumor T cell responses. Nat. Immunol..

[77] Hetfeld B.K., Helfrich A., Kapelari B., Scheel H., Hofmann K., Guterman A., Glickman M., Schade R., Kloetzel P.-M., Dubiel W. (2005). The Zinc Finger of the CSN-Associated Deubiquitinating Enzyme USP15 Is Essential to Rescue the E3 Ligase Rbx1. Curr. Biol..

[78] Nayak A., Glöckner-Pagel J., Vaeth M., Schumann J.E., Buttmann M., Bopp T., Schmitt E., Serfling E., Berberich-Siebelt F. (2009). Sumoylation of the Transcription Factor NFATc1 Leads to Its Subnuclear Relocalization and Interleukin-2 Repression by Histone Deacetylase. J. Biol. Chem..

[79] Xiao Y., Qureischi M., Dietz L., Vaeth M., Vallabhapurapu S.D., Klein-Hessling S., Klein M., Liang C., König A., Serfling E. (2021). Lack of NFATc1 SUMOylation prevents autoimmunity and alloreactivity. J. Exp. Med..

[80] Fang D., Elly C., Gao B., Fang N., Altman Y., Joazeiro C., Hunter T., Copeland N., Jenkins N., Liu Y.-C. (2002). Dysregulation of T lymphocyte function in itchy mice: A role for Itch in TH2 differentiation. Nat. Immunol..

[81] Jin H.-S., Liao L., Park Y., Liu Y.-C. (2012). Neddylation pathway regulates T-cell function by targeting an adaptor protein Shc and a protein kinase Erk signaling. Proc. Natl. Acad. Sci. USA.

[82] Cheng Q., Liu J., Pei Y., Zhang Y., Zhou D., Pan W., Zhang J. (2018). Neddylation contributes to CD4+ T cell-mediated protective immunity against blood-stage Plasmodium infection. PLoS Pathog..

[83] Wang H.X., Pan W., Zheng L., Zhong X.-P., Tan L., Liang Z., He J., Feng P., Zhao Y., Qiu Y.-R. (2020). Thymic Epithelial Cells Contribute to Thymopoiesis and T Cell Development. Front. Immunol..

[84] Weih F., Carrasco D., Durham S.K., Barton D.S., Rizzo C.A., Ryseck R.-P., Lira S.A., Bravo R. (1995). Multiorgan inflammation and hematopoietic abnormalities in mice with a targeted disruption of RelB, a member of the NF-κB/Rel family. Cell.

[85] Akiyama T., Shimo Y., Yanai H., Qin J., Ohshima D., Maruyama Y., Asaumi Y., Kitazawa J., Takayanagi H., Penninger J. (2008). The Tumor Necrosis Factor Family Receptors RANK and CD40 Cooperatively Establish the Thymic Medullary Microenvironment and Self-Tolerance. Immunity.

[86] Burkly L., Hession C., Ogata L., Reilly C., Marconl L.A., Olson D., Tizard R., Gate R., Lo D. (1995). Expression of relB is required for the development of thymic medulla and dendritic cells. Nature.

[87] Naito A., Azuma S., Tanaka S., Miyazaki T., Takaki S., Takatsu K., Nakao K., Nakamura K., Katsuki M., Yamamoto T. (1999). Severe osteopetrosis, defective interleukin-1 signalling and lymph node organogenesis inTRAF6-deficient mice. Genes Cells.

[88] Uchida D., Hatakeyama S., Matsushima A., Han H., Ishido S., Hotta H., Kudoh J., Shimizu N., Doucas V., Nakayama K.I. (2004). AIRE Functions as an E3 Ubiquitin Ligase. J. Exp. Med..

[89] Perniola R. (2018). Twenty Years of AIRE. Front. Immunol..

[90] Lukasiak S., Schiller C., Oehlschlaeger P., Schmidtke G., Krause P., Legler D.F., Autschbach F., Schirmacher P., Breuhahn K., Groettrup M. (2008). Proinflammatory cytokines cause FAT10 upregulation in cancers of liver and colon. Oncogene.

[91] Buerger S., Herrmann V.L., Mundt S., Trautwein N., Groettrup M., Basler M. (2015). The Ubiquitin-like Modifier FAT10 Is Selectively Expressed in Medullary Thymic Epithelial Cells and Modifies T Cell Selection. J. Immunol..

[92] Sanna S., Pitzalis M., Zoledziewska M., Zara I., Sidore C., Murru R., Whalen M.B., Busonero F., Maschio A., Costa G. (2010). Variants within the immunoregulatory CBLB gene are associated with multiple sclerosis. Nat. Genet..

[93] Yokoi N., Fujiwara Y., Wang H.-Y., Kitao M., Hayashi C., Someya T., Kanamori M., Oiso Y., Tajima N., Yamada Y. (2008). Identification and functional analysis of CBLB mutations in type 1 diabetes. Biochem. Biophys. Res. Commun..

[94] Kleine-Eggebrecht N., Staufner C., Kathemann S., Elgizouli M., Kopajtich R., Prokisch H., Lainka E. (2019). Mutation in ITCH Gene Can Cause Syndromic Multisystem Autoimmune Disease with Acute Liver Failure. Pediatrics.

[95] Nguyen T.T.T., Wang Z.-E., Shen L., Schroeder A., Eckalbar W., Weiss A. (2021). Cbl-b deficiency prevents functional but not phenotypic T cell anergy. J. Exp. Med..

[96] Fujiwara M., Anstadt E.J., Khanna K.M., Clark R.B. (2015). Cbl-b-deficient mice express alterations in trafficking-related molecules but retain sensitivity to the multiple sclerosis therapeutic agent, FTY720. Clin. Immunol..

[97] Gómez-Martín D., Ibarra-Sánchez M., Romo-Tena J., Cruz-Ruíz J., Esparza-López J., Galindo-Campos M., Díaz-Zamudio M., Alcocer-Varela J. (2012). Casitas B Lineage Lymphoma b Is a Key Regulator of Peripheral Tolerance in Systemic Lupus Erythematosus. Arthritis Care Res..

[98] Kobashigawa Y., Tomitaka A., Kumeta H., Noda N.N., Yamaguchi M., Inagaki F. (2011). Autoinhibition and phosphorylation-induced activation mechanisms of human cancer and autoimmune disease-related E3 protein Cbl-b. Proc. Natl. Acad. Sci. USA.

[99] Xiao Y., Qiao G., Tang J., Tang R., Guo H., Warwar S., Langdon W.Y., Tao L., Zhang J. (2015). Protein Tyrosine Phosphatase SHP-1 Modulates T Cell Responses by Controlling Cbl-b Degradation. J. Immunol..

[100] Singh T.P., Vieyra-Garcia P.A., Wagner K., Penninger J., Wolf P. (2018). Cbl-b deficiency provides protection against UVB-induced skin damage by modulating inflammatory gene signature. Cell Death Dis..

[101] Loeser S., Loser K., Bijker M.S., Rangachari M., Van Der Burg S.H., Wada T., Beissert S., Melief C.J., Penninger J.M. (2007). Spontaneous tumor rejection by cbl-b-deficient CD8+ T cells. J. Exp. Med..

[102] Sun X., Gao H., Yang Y., He M., Wu Y., Song Y., Tong Y., Rao Y. (2019). PROTACs: Great opportunities for academia and industry. Signal Transduct. Target. Ther..

[103] Cotton A.D., Nguyen D.P., Gramespacher J.A., Seiple I.B., Wells J.A. (2021). Development of Antibody-Based PROTACs for the Degradation of the Cell-Surface Immune Checkpoint Protein PD-L1. J. Am. Chem. Soc..

[104] Yang J., Xu P., Han L., Guo Z., Wang X., Chen Z., Nie J., Yin S., Piccioni M., Tsun A. (2015). Cutting Edge: Ubiquitin-Specific Protease 4 Promotes Th17 Cell Function under Inflammation by Deubiquitinating and Stabilizing RORγt. J. Immunol..

[105] Ruan J., Schlüter D., Wang X. (2020). Deubiquitinating enzymes (DUBs): DoUBle-edged swords in CNS autoimmunity. J. Neuroinflamm..

[106] Harrigan J.A., Jacq X., Martin N.M., Jackson S.P. (2018). Deubiquitylating enzymes and drug discovery: Emerging opportunities. Nat. Rev. Drug Discov..

[107] Brackett C.M., Blagg B.S.J. (2020). Current Status of SUMOylation Inhibitors. Curr. Med. Chem..

[108] Soucy T.A., Smith P.G., Milhollen M.A., Berger A.J., Gavin J.M., Adhikari S., Brownell J.E., Burke K.E., Cardin D.P., Critchley S. (2009). An inhibitor of NEDD8-activating enzyme as a new approach to treat cancer. Nature.

[109] Langston S.P., Grossman S., England D., Afroze R., Bence N., Bowman D., Bump N., Chau R., Chuang B.-C., Claiborne C. (2021). Discovery of TAK-981, a First-in-Class Inhibitor of SUMO-Activating Enzyme for the Treatment of Cancer. J. Med. Chem..

[110] Yoshimura C., Muraoka H., Ochiiwa H., Tsuji S., Hashimoto A., Kazuno H., Nakagawa F., Komiya Y., Suzuki S., Takenaka T. (2019). TAS4464, A Highly Potent and Selective Inhibitor of NEDD8-Activating Enzyme, Suppresses Neddylation and Shows Antitumor Activity in Diverse Cancer Models. Mol. Cancer Ther..

